# The many facets of brain aging

**DOI:** 10.7554/eLife.56640

**Published:** 2020-04-16

**Authors:** Lars Nyberg, Anders Wåhlin

**Affiliations:** 1 Umeå center for Functional Brain Imaging, Umeå UniversityUmeåSweden; 2 Department of Integrative Medical BiologyUmeå UniversityUmeåSweden; 3 Department of Radiation Sciences, Umeå UniversityUmeåSweden

**Keywords:** brain, imaging, UK Biobank, cognition, aging, computational analysis, Human

## Abstract

Applying big-data analytic techniques to brain images from 18,707 individuals is shedding light on the influence of aging on the brain.

**Related research article** Smith SM, Elliott LT, Alfaro-Almagro F, McCarthy P, Nichols TE, Douaud G, Miller KL. 2020. Brain aging comprises many modes of structural and functional change with distinct genetic and biophysical associations. *eLife* **9**:e52677. doi: 10.7554/eLife.52677

We are not all equal when it comes to brain aging: while some people manage to maintain well-preserved cognitive function into old age, others do not (Nyberg et al., 2012). Brain-imaging studies have attempted to capture brain aging by exploring age-related changes to specific structures and different kinds of brain tissue (Good et al., 2001; Walhovd et al., 2005). But a more recent approach has been to use one or more brain-imaging techniques to define a global, single brain-age for each individual (Franke et al., 2010). This estimate is then used to derive a measure called brain-age delta, which represents the gap between the age expected from the brain status and the actual age of the individual. Now, in eLife, Stephen Smith (University of Oxford) and colleagues report a refined brain-age approach that might better represent how aging affects the biological processes of the brain (Smith et al., 2020).

From a sample of 21,407 participants of the UK Biobank study, Smith et al. reported the data of 18,707 individuals over the age of 45 whose brains have been imaged using the same MRI and fMRI protocols. The team then generated 3913 imaging-derived phenotypes: each of the phenotypes represents a different aspect of brain structure or function, such as how specific regions are connected or the structure of certain cortical areas (Figure 1A).

**Figure 1.**
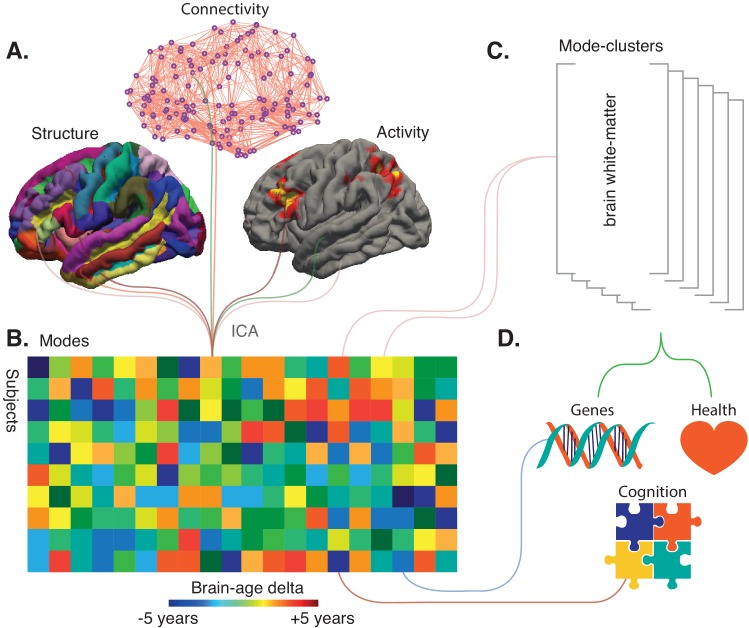
Refining big-data analytic approaches to reveal the many facets of brain aging. (**A**) Smith et al. used a technique called independent component analysis (ICA) to analyze MRI and fMRI data on brain structure, connectivity or activity from more than 18,000 individuals over the age of 45. This enabled them to identify 62 modes. Most of these modes co-varied with age across the sample, thus potentially reflecting biological processes affected by aging. (**B**) Schematic matrix in which each row represents an individual and each column represents a mode. The color scale represents the brain-age delta, the difference between the actual age of the individual and what age would have been expected for this person given the value of the mode. (**C**) The 62 modes can be grouped into six mode-clusters, such as one which captures the microstructure of brain white-matter. (**D**) Smith et al. were able to relate the brain-age deltas for specific modes and the mode-clusters to various phenotypes (for instance health, genetics and cognition).

Further computational analyses were conducted to combine the phenotypes that vary together with age across subjects. In total, 62 of these groups (termed ‘modes’) were established, each potentially representing a biological process affected by aging: for instance, changes in white-matter microstructure could reflect degeneration of axons. These modes were then weighted to describe how strongly each is present in an individual.

The next step was to try to use the modes to assess how an individual would fare in comparison to others, and to examine whether these new measures could correlate with genetic factors. The modes were first grouped to create an ‘all-in-one’ brain-age delta for each participant, a measure that turned out not to be associated with a genetic signature. Next, each specific mode was used to create a brain-age delta that assesses the gap between the participant and the population for this particular mode (Figure 1B). Most of these ‘mode-specific’ brain-age deltas turned out to be associated with the genetic makeup of the individuals. This demonstrates that superimposing distinct brain aging processes in an all-in-one brain-age may obscure biological relations.

While the individual modes might be related to specific biological processes, Smith et al. used data-driven optimization to group the 62 modes into coarser ‘mode-clusters’ (Figure 1C). The six clusters that emerged helped to understand larger patterns of age-related brain changes, and how these relate to other ‘non-imaging’ variables such as health parameters. The mode-cluster that was associated with the greatest aging effect, for example, linked cognitive processing speed with brain patterns like ventricular volume and white-matter microstructure. Diabetes, hypertension, and smoking were all risk factors distributed across mode-clusters, suggesting that different aspects of vascular health influence brain aging through different biological processes.

This work highlights the challenge in determining the optimal balance between integration and diversification in studies of brain aging, where a single brain-age is at the extreme end of integration. An argument in favor of diversification is the fact that, unlike the all-in-one brain-age delta, deltas defined from modes and mode-clusters were associated with genetics (Figure 1D). But what will the optimal ‘unit’ of brain aging turn out to be in the end? Should it be six (as the mode-clusters suggest), 62 (from the analyses of the 3913 imaging-derived phenotypes), or another figure altogether? The relevant number will depend on the type of imaging used in a specific dataset – for example, if it includes both MRI and PET images or MRI alone. Unique brain-age deltas may be revealing a gap between chronological and actual brain age – thus stressing the vast heterogeneity in the older population – but the work by Smith et al. represents the next generation of analytic methods that can help to decode the complexity of brain aging. In the future, genetics and cognition may even be considered in the early stages of analysis, when distinct modes are initially computed.

As imaging data continue to be gathered at a large scale, it is becoming increasingly relevant to examine the complex intrinsic structure of data collected through different imaging methods (Bzdok and Yeo, 2017). Techniques such as the ones used by Smith et al. reduce complexity while respecting the patterns created by actual biological processes, thereby limiting the dilution and potential loss of valuable information. As big-data approaches continue to be refined, they will be able to detect biological processes from brain images, and ultimately these mechanisms might be linked to models of brain aging established at the cellular level.
